# Heat-Resistant Polymer Composites Based on Ethylene Tetrafluoroethylene Mixed with Inorganic Polyoxides

**DOI:** 10.3390/ma14040969

**Published:** 2021-02-18

**Authors:** Alexander Shaulov, Frédéric Addiego, Carlos Eloy Federico, Elena Stegno, Andrei Grachev, Stanislav Patlazhan

**Affiliations:** 1 Semenov Federal Research Center for Chemical Physics, Russian Academy of Sciences, 4, Kosygin Street, 119991 Moscow, Russia; ajushaulov@yandex.ru (A.S.); elena-st@list.ru (E.S.); andrgrachyov@yandex.ru (A.G.); 2 Materials Research and Technology (MRT) Department, Luxembourg Institute of Science and Technology (LIST), ZAE Robert Steichen, 5 Rue Bommel, L-4940 Hautcharage, Luxembourg; carloseloy.federico@list.lu

**Keywords:** ethylene tetrafluoroethylene, aluminum polyphosphate, metakaolin, polymer composite, characterization

## Abstract

This pilot study aimed at investigating an alternative to irradiation-crosslinking to increase the structural stability of ethylene tetrafluoroethylene (ETFE), by mixing this polymer matrix with polyoxides. The latter consisted of aluminum polyphosphate (AP) having a flow temperature near to that of ETFE to facilitate melt-mixing by extrusion, and rigid fillers of metakaolin (MK). It was found that the ETFE/AP/MK composite with the formulation 60/20/20 (wt %) exhibited the most relevant properties. Indeed, when comparing this composite with neat ETFE, the structural stability was improved until 120 °C, the onset temperature of degradation passed from 381.5 to 459.4 °C, the elastic modulus evolved from 0.4 GPa to 1.6 GPa, and the tensile strength increased from 23 to 27 MPa. The results were briefly discussed based on a potential interaction between the polyoxides and the polymer matrix and synergistic effect between the two polyoxides.

## 1. Introduction

Polymer-based composites are materials of choice for manifold applications including automotive, aircraft, and space industries. Their advantages are defined by high mechanical properties at relatively low mass per unit volumes, providing high productivity at reduced power consumption. The binders commonly used for such applications are the high-performance grades of thermoplastic or thermosetting polymers having high thermal resistance and withstanding exposure to many thermal cycles in space. Fluoropolymers seem suitable from this point of view, however, most of them cannot be manufactured by melt processing because of their high viscosity. To address this issue, a copolymer of ethylene and tetrafluoroethylene, named ethylene tetrafluoroethylene (ETFE), has been developed to benefit both from the advantage of fluoropolymer concerning thermal resistance, and of thermoplastics concerning melt-processing ability.

One of the most important space applications of ETFE is electrical cable jacketing, the material requiring an irradiation-crosslinking treatment step to ensure structural stability in the space environment [1]. Compared to untreated ETFE, crosslinked ETFE exhibited an increase of tensile modulus and strength with increasing irradiation dose, while thermally-setting the physical structure of the material (preventing softening/melting and crystallization) [2]. However, irradiation crosslinking has adverse effects as decreasing elongation at break [2], decreasing degradation temperature [3], and initiating material degradation [3]. Furthermore, this treatment is complex since it relies on many parameters such as temperature, material atmosphere, irradiation dose, and most of the time requires stabilization post-treatment to avoid further degradation.

It is proposed here to explore an alternative method to enhance the structural stability of ETFE for space application by dispersing inorganic polyoxides within this matrix. These inorganic polyoxides are rigid-chain nonflammable thermoplastics and should be selected with a flow temperature close to that of ETFE to facilitate melt-processing. Blends of inorganic thermoplastic polyoxides with thermoplastic polymers were successfully developed in the case of commodity and engineering plastics as polyethylene and polyamide [4,5,6], while blends of polyoxides with high-performance thermoplastics have been little developed yet. In this last case, poly(vinylidenedifluoride) was blended with oxyfluoride providing a composite with a limited drop of the elongation at break and increase Young’s modulus compared to the neat polymer appearing suitable, but the thermal resistance of the composite was not evaluated [7]. It was explained that the ability of the polyoxides and thermoplastic matrix to pass into a highly elastic state during an extrusion process is accompanied with a nano- and molecular-level of mixing between the two components [8,9]. Accordingly, the resulting composites were proved to exhibit a high degree of filling (the model of “softening filler”) and a regulation of the crystallinity, supramolecular structure [5], and rheological properties [10,11,12]. Under certain conditions, the formation of inorganic microfibers was also noted [5,11,12]. Some blends of polyoxides with poly(ethylene) and poly(vinyl alcohol) were demonstrated to enhance the heat resistance of the neat polymer matrices [13,14].

The objective of this pilot study is to screen new formulations of ETFE with two kinds of inorganic polyoxides that are aluminum polyphosphate and/or metakaolin to enhance the structural stability of ETFE. To this end, small batches of ETFE-based materials were prepared by melt-processing and their microstructural, thermal and mechanical properties were carefully assessed. Aluminum polyphosphate was synthesized at different temperatures and thermally-analyzed to choose the most appropriate flow temperature for being mixed with ETFE. The idea here was to get a softening of both ETFE and aluminum polyphosphate to create a nano- and possibly a molecular level of mixing between those two components. Concerning metakaolin, it was used as a chemically active component that can be dissolved in aluminum polyphosphate. A potential synergistic effect between those two polyoxides can be expected on the thermal resistance of the composite. As a comparison, the same characterization was done on ETFE filled with conventional inorganic micro-particles of titanium dioxide.

## 2. Materials and Methods

**Figure 1:**
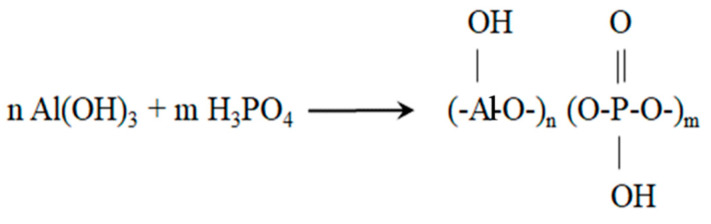
The scheme of synthesis of aluminophosphate oligomers by the reaction of aluminum hydroxide with phosphoric acid.

The raw materials used in this study were (i) an ETFE grade reference Tefzel 207 from DuPont (Wilmington, DE, USA) (density 1.7 g/cm^3^, softening temperature T_s_ of 255 °C, flow temperature T_f_ of 275 °C, (ii) a 55% aqueous solution of aluminophosphate oligomers (Al/P = 1/2) supplied by VZHR trading house (Voskresensk, Russia), (iii) metakaolin (MK) solid fillers (aluminum silicate, Al_2_O_3_2SiO_2_, specific surface area SSA of 2 m^2^/g) supplied by Synergo (Chelyabinsk, Russia), and (iv) titanium dioxide (TiO_2_) solid fillers provided by DuPont Titaniun Technologies (New Jonsonville, TN, USA), (average particle size 5 μm). The original aluminophosphate oligomers were synthesized by reaction of aluminum hydroxide with phosphoric acid at 900 °C. The reaction scheme is represented in Figure 1. Heating an aqueous solution of aluminophosphate oligomers at a constant temperature ranging from 130 to 280 °C stimulated polycondensation processes that led to synthesis of aluminum polyphosphate (AP). Different temperatures were used to produce AP grades with different thermomechanical properties.

**Figure 2:**
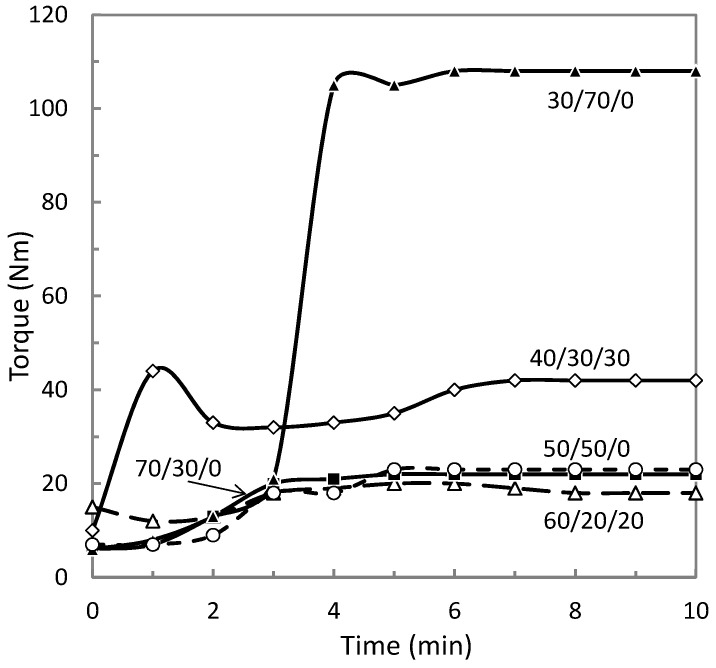
Torque of the screw extruder as a function of time for different ETFE/AP/MK compositions in wt %. AP was synthesized at 270 °C. ETFE, ethylene tetrafluoroethylene; AP, aluminum polyphosphate; MK, metakaolin.

The composite melt-processing was carried out on a Haake twin-screw micro-extruder (Thermo Fischer Scientific, Karlsruhe, Germany) with a volume of 7 cm^3^ at a temperature of 285 °C, a screw rotation speed of 50 rpm, and a mixing time of 15 min (see Figure 2). A batch of several tens of grams was prepared per formulation.

Samples for physico-mechanical testing were obtained by injection molding at a melt temperature of 285 °C, a pressure of 8 bar applied during 10 s, and a mold temperature of 80 °C. The following material compositions ETFE/AP/MK/TiO_2_ were processed: 30/70/0/0, 50/50/0/0, 70/30/0/0, 60/20/20/0, 40/30/30, 55/0/45/0, and 65/0/0/35 (wt %).

The material internal structure was analyzed in 3D employing a micro-computed x-ray tomograph (µCT) EasyTom 160 from RX Solutions (Chavanod, France). Specimen were scanned at a voltage of 70 kV and a current of 35 µA, utilizing a micro-focused tube equipped with a tungsten filament (Hamamatsu Photonics, Hamamatsu, Japan). The source-to-detector distance (SDD) and the source-to-object distance (SOD) were set to have a voxel size of 6 µm. The recorded slices were treated with the software X-Act 64 from RX Solutions (version 2020, Chavanod, France) to reconstruct the volume after inherent corrections (geometrical and ring artefact corrections). Then, the data were treated and analyzed with the software Avizo provided by Thermo Fischer Scientific (version 2019.1, Waltham, MA, USA) enabling to threshold the intensity histogram for segmentation, and hence, to extract and analyze the objects of interest (pores and polyoxides within ETFE). In particular, the software provided their equivalent diameter, number per unit volume, and volume fraction.

The thermal transitions of the materials were analyzed with a differential scanning calorimeter DSC 3+ from Mettler-Toledo (Greifensee, Switzerland). In particular, samples with a weight comprised between 5 and 10 mg were sealed in aluminum pans and submitted to a first heating stage from 25 to 300 °C to erase thermal history, then to a cooling stage from 300 to 25 °C, and last to a second heating stage from 25 to 300 °C enabling the capture of intrinsic thermal properties. Differential scanning calorimetry (DSC) testing was done with heating and cooling rates set to 10 °C/min, and with a nitrogen gas purge. From the obtained thermograms, the melting temperature *T_m_* and the melting enthalpy $\Delta H_{m}$ were determined from the first and second heating stages, while the crystallization temperature *T_c_* was assessed from the cooling stage. The degree of crystallinity $X_{c}$ (in wt %) was estimated as:

(1) $$X_{c}\left(\%\right)=\frac{\Delta H_{m}}{\Delta H_{m}^{o}}\cdot \frac{100}{f_{ETFE}}$$

where $\Delta H_{m}^{o}$ represented the heat of fusion for 100% crystalline ETFE, which was taken equal to 113.4 J/g [15], and $f_{ETFE}$ was the ETFE weight fraction.

The thermomechanical properties of the samples were determined on a Netzsch TMA 402 instrument (Selb, Germany) with a standard load of 500 kN. This equipment enabled to determine the dimensional changes in the composites as a function of temperature. 

Thermogravimetric analyses were performed on a Netzsch DSK Jupiter STA 449 F3 instrument (Selb, Germany) from 20 to 500 °C. A stream of air at the atmospheric pressure and a speed of 30 ml/min was used. The heating rate was set to 10 °C/min.

The tensile behavior of the ETFE-based composites was estimated in tension by using a testing machine LRX plus from Lloyd Instruments (Fareham Hampshire, UK) were carried out on standard specimen at 20 °C and a tensile deformation rate of 100 mm/min. Based on the stress–strain curves, the tensile modulus, strength, and elongation at break were measured at different weight fractions of aluminum polyphosphate, metakaolin and titanium dioxide fillers.

## 3. Results and Discussion

### 3.1. Processability

The extrusion torque of ETFE composites was monitored as a function of time, as depicted in Figure 2. Such a monitoring may provide new features about the processing of this new ETFE-based materials. The components of the composite were introduced into the extruder sequentially starting with the fluoropolymer. The AP tested in this part was synthesized at a temperature of 270 °C since it is close to ETFE flow temperature which provides the best conditions for producing more homogeneous specimens (see Section 3.2 and Section 3.3). The highest extrusion duration must be chosen to maximize the distribution and dispersion of the polyoxides within the polymer matrix, while at the same time degradation of the polymer matrix may not be started, meaning that the extrusion torque should not to decrease. Accordingly, 10 min was chosen as the mixing time for all the compositions. Another important information provided by these measurements is the highest volume fraction of the polyoxide AP that can be mixed with ETFE using our equipment, corresponding to the ETFE/AP composition 30/70. Indeed, such a composition was characterized by the highest attainable torque of the extruder that was equal to 110 Nm. Considering an AP density of 2.5 g/cm^3^, this maximum weight fraction of 70 wt % corresponds to a volume fraction of 61.3 vol%, appearing higher than the maximum content in conventional micro-metric fillers that can be mixed with a polymer matrix (about 50 vol% [16]). Therefore, polyoxides offer more flexibility in term of formulation compared to conventional micro-metric fillers, enabling to potentially discover new composite performance.

### 3.2. Thermal Properties

**Figure 3:**
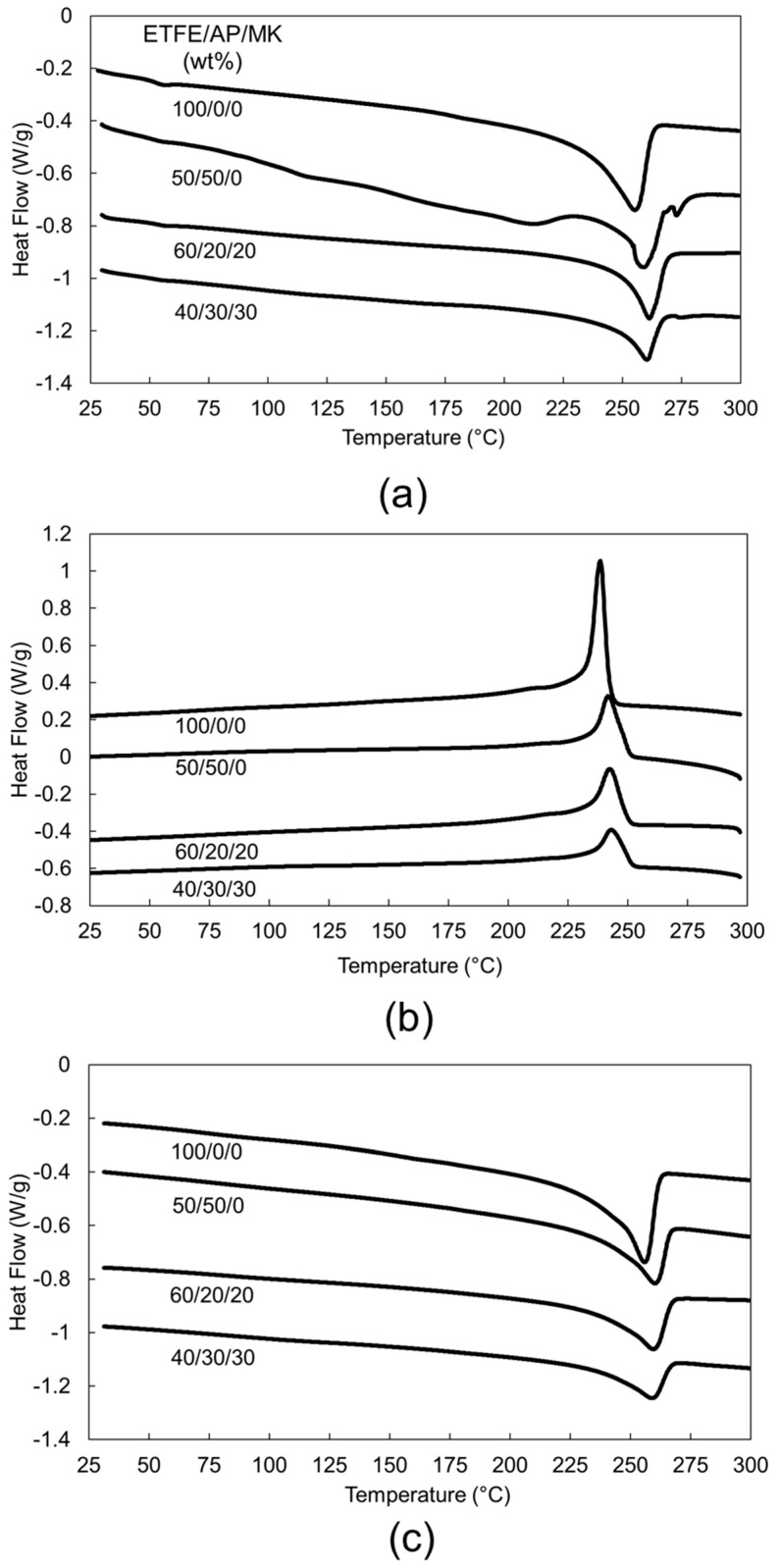
Differential scanning calorimetry (DSC) curves of selected ETFE/AP/MK composites, (**a**) first heating stage, (**b**) cooling stage, and (**c**) second heating stage.

The DSC curves of the materials are plotted in Figure 3, while the extracted data is reported in Table 1. Although the first heating stage was done to erase the thermal history of the materials, it should be described since it reflects the effective thermal properties of the materials prior to thermomechanical and tensile testing. In this first heating stage, an endothermic peak is noted at around 56 °C, which can be attributed to a crystalline phase transformation occurring for ETFE. It is due to a transition from the orthorhombic phase to the hexagonal phase and is named α’ relaxation [17]. All the materials exhibited the expected thermal behavior of ETFE, with an endothermic melting peak during the heating stages, and a crystallization exothermic peak during the cooling stage. Nevertheless, in the case of ETFE/AP/MK (50/50/0) composite, the DSC thermogram of the first heating stage exhibits a non-expected behavior with numerous small endothermic peaks superposed to the ETFE thermal behavior. Note that as for previous works, the glass transition (α relaxation) was not observable by DSC in the case of ETFE [18]. By dynamic mechanical analysis, it was reported that the glass transition temperature of ETFE was about 100 °C [18,19].

The origin of those additional peaks, which disappear during the subsequent cooling and the second heating stages, may be explained by the presence of residual unreacted products of AP polymerization. The latter may totally react during this first heating stage since the second heating stage does not exhibit the corresponding peaks. One important consequence of those peaks is that the melting enthalpy of ETFE cannot be calculated with precision during the first heating stage. When comparing neat ETFE with ETFE composites, the melting temperature, the crystallinity and the crystallization temperature increased significantly, indicating a potential interaction between ETFE and the polyoxides. In the case of ETFE mixed with AP, the polyoxide may act as a nucleating agent for ETFE improving its crystallization. It is not clear yet if MK also acts as a nucleating agent since we did not test ETFE filled with MK.

In the literature, numerous possible nucleating agents have been reported for fluoropolymers, including phosphonium salts, pyridinium salts, pyrrolidinium salts, sulfonium, sulfonate, phosphonate, and boron nitride [20] but to our best knowledge aluminum polyphosphate has never been mentioned as a nucleating agent for fluoropolymers. It was explained that due to the fluorine, fluoropolymers are negatively charged at their surface, and hence, a positively charged surface is required for the crystallization [20]. Accordingly, an electrostatic interaction is involved between the nucleating agent and ETFE, which may be the case for AP.

However, in general, only a weak fraction of nucleating agent is needed for the crystallization of a polymer, for example 0.5 wt % in the case of boron nitride for a fluoropolymer [20]. In the case of the composite ETFE/AP/MK (40/30/30), the nucleating effect of AP and/or MK is partially counterbalanced by the important viscosity induced by the very high filler content. Therefore, this material has a lower increase of crystallinity, melting temperature and crystallization temperature compared to the two other composite formulations.

**Figure 4:**
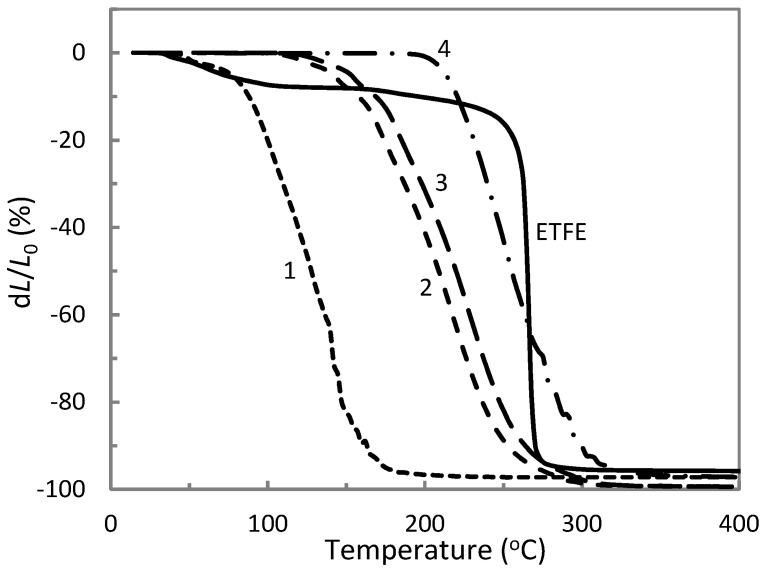
Thermomechanical curves of the neat ETFE and aluminum polyphosphate synthesized at 130 °C (1), 260 °C (2), 270 °C (3) and 280 °C (4).

The comparison of the thermomechanical curves of AP synthesized at different temperatures and neat ETFE are depicted in Figure 4. It is noted that increasing heating temperature resulted in a shifting of both the softening temperature *T_s_* and the flow temperature *T_f_* of the obtained aluminum polyphosphates towards larger values. The data obtained from Figure 4 is collected in Table 2. The results indicate a growth of the molecular mass of aluminophosphate oligomers due to the polycondensation processes [21,22]. Among the obtained AP materials, we selected the one synthesized at 270 °C since it possesses a flow temperature close to that of ETFE. This feature provides the best conditions for the composite melt-processing at 285 °C resulting in more homogeneous specimens (see Section 3.3). Concerning ETFE, we can note a softening until 120 °C that can be attributed to the glass transition of the material named α relaxation, as shown in previous works by dynamic mechanical analysis [18,19]. The α’ relaxation noted in DSC curves (Figure 3) was not observed in the thermomechanical curves, probably due to a non-significant change of material hardness.

**Figure 5:**
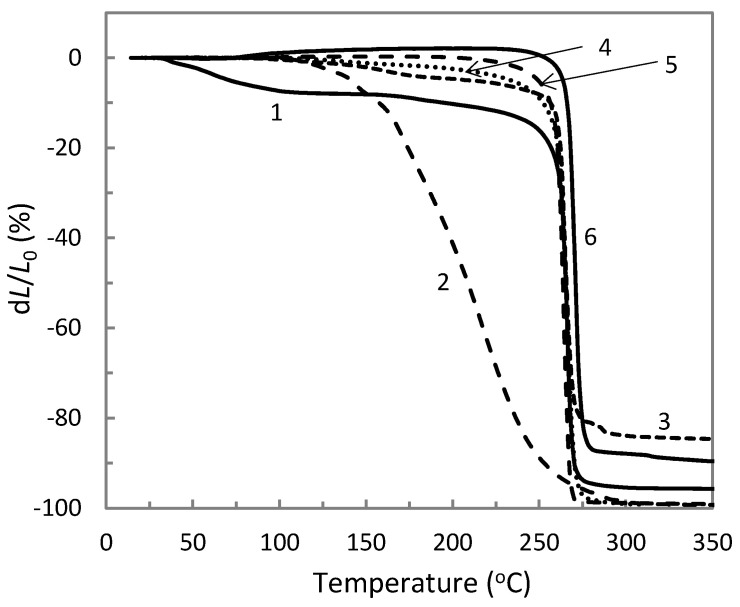
Thermomechanical curves of neat ETFE (1), AP synthesized at 270 °C (2), ETFE/AP composites with the compositions 30/70 (3), 50/50 (4), 70/30 (5), and ETFE/TiO_2_ with the composition 65/35 (6) (wt %). In the case of the composites AP was also synthesized at 270 °C.

The thermomechanical curves of ETFE composites in Figure 5 show a first important result that is the disappearance of the glass transition phenomenon. As a result, ETFE composites exhibited higher structural stability than neat ETFE at until 120 °C, which is suitable to develop an alternative to irradiation-crosslinking. This finding is not influenced by the type of fillers since both ETFE/AP and ETFE/TiO_2_ presented the same behavior. It is thought that the glass transition of highly filled ETFE is shifted to higher temperatures due to the molecular motion hindering by the fillers and the higher crystallinity (Table 1), and is less intense due to the reduction of the matrix and amorphous phase fraction so that it is no more visible on the thermomechanical curves. Considering the average absolute deviation of 5 °C, the softening temperature of the composites did not differ from that of neat ETFE that was equal to *T_s_* = 255 °C. This finding highlights no molecular interaction between ETFE and AP, since the softening of the composites does not occur between that of neat ETFE and AP (Table 2). This lack of molecular interaction is further confirmed by the curve of the composite ETFE/AP 30/70. Indeed, for this composite formulation a small softening phenomenon starting at 160 °C was noted, corresponding to the softening of the polyoxide, while the main softening was observed at 255 °C attributed to the ETFE matrix. Therefore, the molecular interaction noted by DSC was not detected by these thermomechanical measurements.

**Figure 6:**
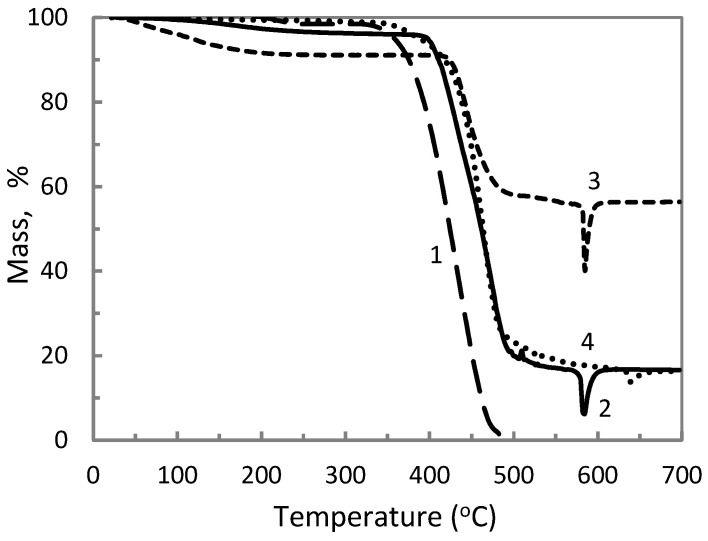
Thermogravimetric curves of ETFE/AP composites with the following compositions: 100/0 (1), 70/30 (2), 30/70 (3), and 50/50 (4) (wt %).

**Figure 7:**
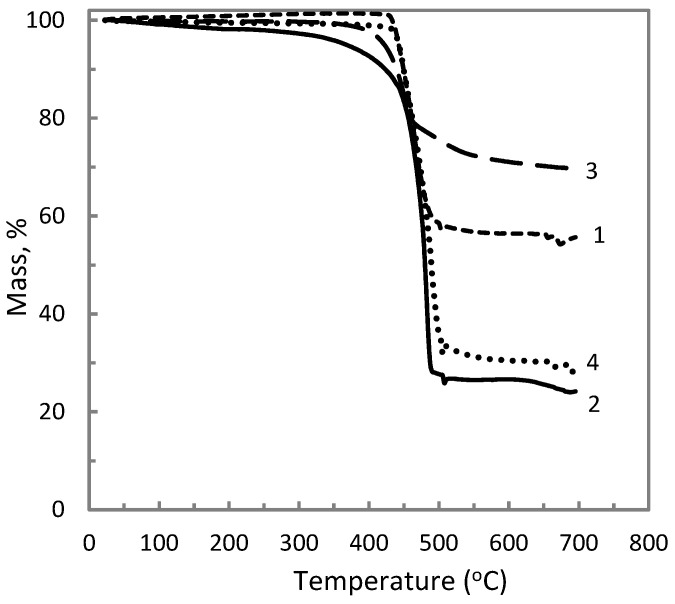
Thermogravimetric curves of ETFE/AP/MK/TiO_2_ composites with the following compositions: 40/30/30/0 (1), 60/20/20/0 (2), 55/0/45/0 (3), and 65/0/0/35 (4) (wt %).

The effect of aluminum polyphosphate, metakaolin and titanium dioxide on the thermal properties of the ETFE polymer was further studied by thermogravimetric analysis in the case of various compositions. The change in temperature at the onset of mass loss in ETFE/AP mixtures is presented in Figure 6 and for mixtures with the fillers MK and TiO_2_ in Figure 7. An increase in the temperature onset of weight loss is observed when mixing ETFE with AP, and or MK, or TiO_2_. All the data on the heat resistance of the obtained composites is listed in Table 3. In general, conventional fillers in polymer matrix enhance thermal resistance by acting as a physical barrier to oxygen, and/or acting as a radical scavenger, and/or hindering the transport of degradation products. These effects depend on the chemical nature of the fillers and are more efficient in case of a high aspect ratio and dispersion state of the filler. Phosphates are the known fire retardants of hydrocarbons which are the part of ETFE copolymer chain [23].

It is hence possible that polyoxides act as conventional fillers, but a more detailed study is required to identify the mechanisms responsible for the improved thermal resistance. The highest increase of the temperature onset of weight loss compared to neat ETFE is noted for the composition ETFE/AP/MK (60/20/20), which is equal to 77.9 °C, indicating a possible synergistic effect of AP and MK. The identification of the underlying mechanisms requires additional investigations.

### 3.3. Microstructure

**Figure 8:**
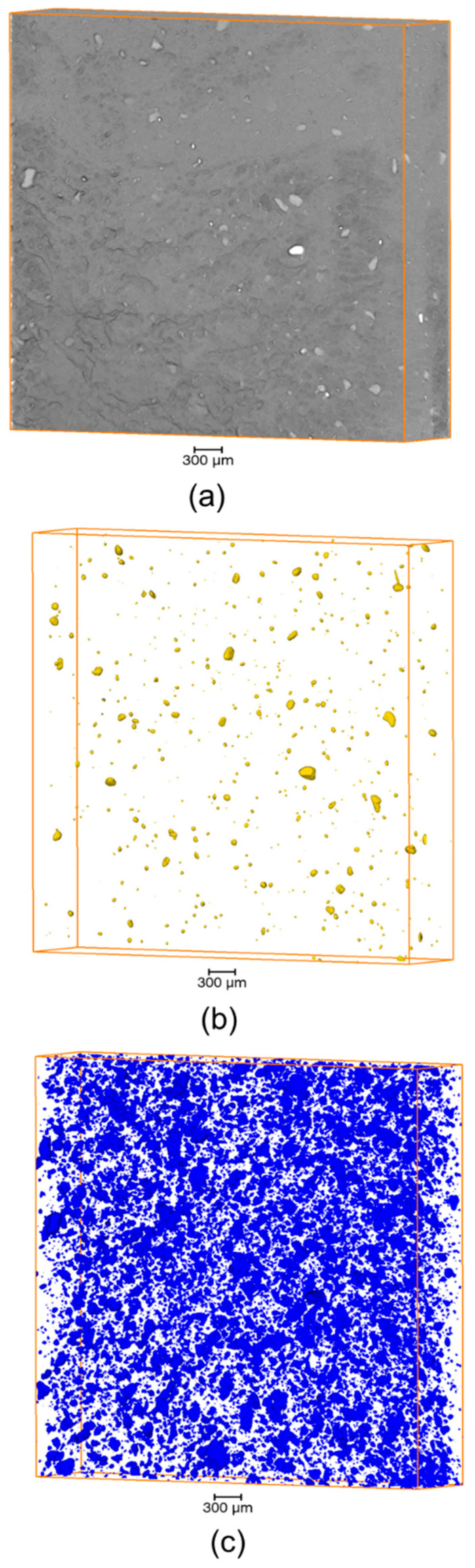
Volume rendering (5 mm × 5 mm × 1 mm) obtained by µCT of ETFE/AP/MK (60/20/20): (**a**) overall composite visualization; (**b**) AP object visualization; (**c**) MK object visualization. AP was synthesized at 270 °C.

The internal microstructure of the materials has been inspected by µCT. An example of volume rendering is provided in Figure 8 concerning the composite ETFE/AP/MK (60/20/20) with AP synthesized at 270 °C. The overall composite visualization (Figure 8a) exhibited a matrix in which were distributed two kinds of objects appearing with two different intensities. After segmentation by intensity histogram thresholding, it was possible to isolate the two families of objects that were the AP phase appearing as a rigid filler (Figure 8b) and the rigid MK fillers (Figure 8c). Above the resolution of the µCT (>6 µm), these two objects were homogeneously distributed within the scanned volume since there is no region where these objects were not present. However, the spatial distribution of the objects below the µCT resolution (<6 µm) cannot be observed.

The mean equivalent diameter of these two objects was in the range between 20 and 40 µm, while their number per mm^3^ strongly depended on the material composition (Table 4). Compared to their theoretical volume fraction (calculated based on an approximative density of 2.5 g/cm^3^ for both AP and MK) a very low volume fraction of objects was detected, indicating that most of the polyoxide phase was not detected by µCT. The majority of AP and MK objects may be below the µCT resolution (6 µm). The number of detected MK objects per mm^3^ and their volume fraction was much higher than those of AP objects for the same weight fraction. For example, in the composite formulation ETFE/AP/MK (60/20/20), the detected volume fraction of AP was 0.11 vol%, while that of MK was 3.02 vol% (factor of 27.4).

This finding may be due to mixing of the components that may conduct to a nano- and possibly to a molecular level of mixing between AP, MK and ETFE, resulting in a limited number of AP and MK objects at the micron-scale. In particular, the addition of metakaolin to ETFE may result in a fine dispersion as MK is known to exhibit a lamellar structure that can be exfoliated [24]. Note that in the case of the composite ETFE/AP/MK (40/30/30), the presence of pores was noted, which is not suitable to ensure good mechanical properties. Such pores may be due to a non-optimal injection process of this composite exhibiting a very high viscosity due to its high fraction of polyoxides.

### 3.4. Mechanical Properties

The Young’s modulus E, tensile strength σ, and elongation at break ε of neat ETFE and its composites with aluminum polyphosphate, metakaolin and titanium dioxide fillers were measured. The results are summarized in Table 5.

In all the composites, an increase in E and a decrease of σ and ε were noted in comparison with neat ETFE. It is observed that the addition of 30 wt % of aluminum polyphosphate resulted in a weak variation of Young’s modulus and tensile strength but to an important decrease in the elongation at break of ETFE/AP composites. On the contrary, the addition of 35 wt % of TiO_2_ to ETFE led to a drastic increase of the elastic modulus reaching 1.1 GPa and to the highest drop of strength and elongation at break reaching 8 MPa and 1.6%, respectively. This finding is probably due to the much higher stiffness of TiO_2_ fillers and weaker adhesion with ethylene tetrafluoroethylene matrix as compared with the polyoxides.

**Figure 9:**
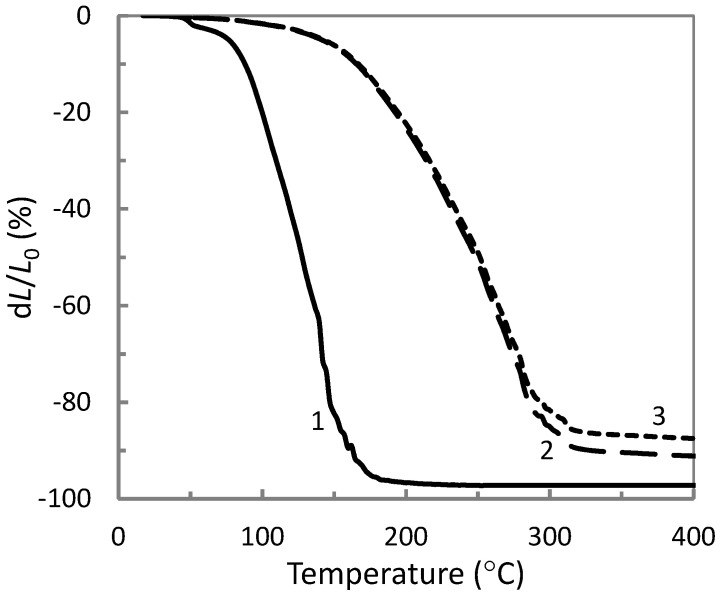
Thermomechanical curves of neat AP synthesized at 130 °C (1) and AP/MK composites with compositions 70/30 (2), 50/50 (3).

The composition of ETFE with metakaolin improved the tensile strength and elongation at break and increased significantly the Young’s modulus. The obtained results indicate a better adhesion of metakaolin to ETFE compared to aluminum polyphosphate. Interestingly, the combination of aluminum polyphosphate with metakaolin in ETFE matrix was also characterized by the relatively high tensile strength that exceeded that of the fluorinated polymer for the ETFE/AP/MK composition of 60/20/20. In this case, Young’s modulus even increased while the elongation at break was of 28.5% indicating a certain level of ductility. Those mechanical properties are in line with the ones of irradiation-crosslinked ETFE [2]. Further increase in both AP and MK volume fractions led to a drop of the elongation at break but still preserve a high tensile modulus and strength. These observations indicate that the presence of a certain amount of metakaolin can improve the mechanical properties of fluoro/inorganic polymer blends. However, a too high fraction of aluminum polyphosphate and metakaolin leads to poor elongation at break. These results do not seem trivial since the absence of loss of the tensile strength of the composites has been achieved at a high total concentration of the fillers, while their elastic moduli are much larger than that of the neat ETFE matrix. This effect is impossible to reach while using traditional mixtures with dispersed fillers. We could suppose that the observed results can be a consequence of the chemical interaction between the dispersed metakaolin and aluminum polyphosphate [22] resulting in the formation of a new inorganic polymer with T_s_ and T_f_ values significantly higher than those of aluminum polyphosphate even synthesized at low temperature (130 °C) (see Figure 9). This is indirectly confirmed by the fact that AP/MK compositions presented thermomechanical curves close to those of thermoplastics. This sort of behavior is demonstrated in Figure 9 by the example of AP synthesized at 130 °C. Note that chemical interaction between AP and MK can also explain their synergistic effect observed in TGA curves (Table 3).

## 4. Conclusions

A novel heat-resistant polymer-based composite has been processed for the first time. It was obtained from the melt-processing of an ethylene tetrafluoroethylene (ETFE) matrix with a thermoplastic grade of aluminum polyphosphate (AP) obtained from the polycondensation of aluminophosphate oligomers and/or rigid fillers of metakaolin (MK). In this pilot study, the influence of the type of inorganic phase and its concentration was investigated on the thermal, thermomechanical and mechanical properties of the composite. The main finding indicates that the addition of AP and/or MK to the ETFE matrix significantly increased the structural stability at moderate temperatures (until 120 °C) and the thermal degradation onset temperature of ETFE, while mechanical properties were affected in different manners. In general, the tensile modulus increased and the elongation at break decreased when mixing ETFE with AP and/or MK, while the tensile strength increased or decreased depending on the formulation. The most promising results were obtained in the case of ETFE/AP/MK 60/20/20 (wt %) exhibiting an increase of the onset degradation temperature by 20.4%, an increase of elastic modulus by 300%, and an increase of the tensile strength by 17.4% compared to neat ETFE while exhibiting a certain ductility (28.5%). Both AP and MK were detected to have effective interactions with the ETFE matrix. AP was proved to enhance the crystallization of ETFE and increase the crystallization temperature of the matrix (molecular interaction). MK was proved to have good mechanical adhesion with the ETFE matrix (microscopic reinforcing agent). µCT revealed that AP was almost no present at the micro-scale contrary to MK fillers, indicating possibly a nanoscale or molecular level of mixing between AP and ETFE. It may be suggested that a fine dispersion of the AP fire retardant allows increasing contact number of phosphate groups with hydrocarbon part of ETFE copolymer, thereby improving its thermal resistance. AP and MK can also interact having a synergistic effect concerning thermal resistance. 

This pilot study has shown the potential of mixing fluoropolymers with polyoxides (AP and MK) as an alternative to irradiation-crosslinking. Indeed, the structural stability of ETFE is improved at moderate temperatures, the onset temperature of degradation is increased, elastic modulus and tensile strength are improved, while ductility is still present. However, for a better understanding of the underlying mechanisms, a careful identification of the initial physical and chemical structure of the two polyoxides and a careful study of their potential interaction is required.

## Figures and Tables

**Table 1:** DSC analysis of selected ETFE/AP/MK composites (n.m. stands for not measurable).

ETFE/AP/MK Composition (wt %)	First Heating		Cooling	Second Heating	
	*T_m_* (°C)	*X_c_* (wt %)	*T_c_* (°C)	*T_m_* (°C)	*X_c_* (wt %)
100/0/0	255.3 ± 0.4	57.7 ± 2.8	238.8 ± 0.0	255.7 ± 0.1	56.2 ± 4.0
50/50/0	260.8 ± 2.1	n.m.	241.9 ± 0.2	259.9 ± 0.3	70.0 ± 7.6
60/20/20	261.2 ± 0.4	70.3 ± 1.3	242.7 ± 0.3	259.2 ± 0.2	70.2 ± 2.2
40/30/30	259.8 ± 0.6	65.9 ± 3.7	243.3 ± 0.1	258.5 ± 0.2	62.5 ± 1.0

**Table 2:** Softening temperature (*T_s_*) and flow temperatures (*T_f_*) of ETFE and AP synthesized at different temperatures (average absolute deviation of 5 °C for each *T_s_* and *T_f_*).

Materials	ETFE	AP (130 °C)	AP (260 °C)	AP (270 °C)	AP (280 °C)
*T_s_* (°C)	255	80	145	160	210
*T_f_* (°C)	275	170	260	280	300

**Table 3:** Heat resistance of the composites depending on the content of inorganic components.

ETFE/AP/MK Composition (wt %).	Total Filler Content (wt %)	T_onset_ (°C)
100/0/0	0	381.5
70/30/0	30	406.8
50/50/0	50	427.6
60/20/20	40	459.4
40/30/30	60	438.2
55/0/45	45	420.0

**Table 4:** Analysis of the polyoxides and pores in selected ETFE/AP/MK composites from µCT measurements.

ETFE/AP/MK Composition (wt %)	Object of Interest	Mean Equivalent Diameter (µm)	Number of Objects per mm^3^	Volume Fraction (vol%)	Theoretical Volume Fraction (vol%)
50/50/0	AP	39.6	178.7	1.10	40.00
60/20/20	AP	30.2	26.2	0.11	15.60
	MK	39.6	390.9	3.02	15.60
40/30/30	AP	23.5	21.0	0.03	25.20
	MK	30.3	1015.7	3.89	25.20
	Pores	212.0	5.6	6.36	0.00

**Table 5:** Mechanical properties of the composites (the average standard deviation was ±12%).

ETFE/AP/MK Composition (wt %)	*E* (GPa)	σ (MPa)	ε (%)
100/0/0	0.4	23	185.1
70/30/0	0.5	21	14.0
50/50/0	0.6	17	4.5
60/20/20	1.6	27	28.5
40/30/30	1.7	21	2.6
55/0/45	1.2	30	49.0

## Data Availability

The data presented in this study are available on request from the corresponding authors.
